# The combination effect of sodium butyrate and 5-Aza-2'-deoxycytidine on radiosensitivity in RKO colorectal cancer and MCF-7 breast cancer cell lines

**DOI:** 10.1186/1477-7819-7-49

**Published:** 2009-05-21

**Authors:** Hang Joo Cho, Sin Young Kim, Kee Hwan Kim, Won Kyung Kang, Ji Il kim, Seong Tack Oh, Jeong Soo Kim, Chang Hyeok An

**Affiliations:** 1 Department of Surgery, Uijongbu St Mary's Hospital, College of Medicine, The Catholic University of Korea, South Korea; 2 Department of Surgery, Kangnam St Mary's Hospital, College of Medicine, The Catholic University of Korea, South Korea

## Abstract

**Background:**

The overall level of chromatin compaction is an important mechanism of radiosensitivity, and modification of DNA methylation and histone deacetylation may increase radiosensitivity by altering chromatin compaction. In this study, we investigated the effect of a demethylating agent, a histone deacetylase(HDAC) inhibitor, and the two agents combined on radiosensitivity in human colon and breast cancer cell lines.

**Methods:**

In this study, we used RKO colorectal cancer cell line and MCF-7 breast cancer cell lines and normal colon cell lines. On each of the cell lines, we used three different agents: the HDAC inhibitor sodium butyrate(SB), the demethylating agent 5-Aza-2'-deoxycytidine(5-aza-DC), and radiation. We then estimated the percentage of the cell survival using the XTT method and experimented to determine if there was an augmentation in the therapeutic effect by using different combinations of the two or three of the treatment methods.

**Results:**

After treatment of each cell lines with 5-aza-DC, SB and 6 grays of radiation, we observed that the survival fraction was lower after the treatment with 5-aza-DC or SB than with radiation alone in RKO and MCF-7 cell lines(p < 0.001). The survival fraction was lowest when the two agents, 5-aza-DC and SB were combined with radiation in both RKO and MCF-cell lines.

**Conclusion:**

In conclusion, 5-aza-DC and SB can enhance radiosensitivity in both MCF-7 and RKO cell lines. The combination effect of a demethylating agent and an HDAC inhibitor is more effective than that of single agent treatment in both breast and colon cancer cell lines.

## Background

Epigenetics is an important intracellular procedure that can change the genetic information of the cells that is transmitted during cell division without changing the sequences of the DNA bases [1]. Of the mechanisms of epigenetics, methylation of DNA and histone alteration are related to carcinogenesis.

DNA methylation is carried out by DNMT (DNA methyltransferase), usually when a methyl group is added to the cytosine residue of a CpG island, which is a group of repeated CpG sequences [2]. Aberrant methylation of DNA has an important role in controlling genes and epithelial carcinogenesis. When methylation of the CpG island which is at the promoter region of the genetic sequence, occurs the transcription of the gene is suppressed. If hypermethylation occurs at the promoter region of the tumor suppressor genes, transcription is inhibited, which results in the loss of the function of the gene. This functional loss brings about an inability to suppress cell proliferation, which can lead to carcinogenesis [2-4].

Histone alteration is another epigenetic mechanism of regulating transcription. The histone octamer consists of a core, which is encircled by double stranded DNA to form a nucleosome. Two enzymes are associated with histone deacetylation – histone acetyltransferase and histone deacetylase(HDAC) [5]. HDAC takes part in carcinogenesis by regulating cell cycle progression, mitosis, and transcription of genes that participate in apoptosis. Recently a great deal of research has been carried out focusing on the inhibition of HDAC [6].

The biggest difference between the mechanisms of epigenetics and genetics is that epigenetics can be reversed by using certain chemical substances [1]. Also, there have been recent reports that histone deacetylation, combined with DNA methylation of tumor suppressor genes, can suppress the function of genes [7-11]. According to this mechanism, the combination of demethylating agents and HDAC inhibitors as an ideal epigenetics treatment modality may bring about good results.

Recently, there has been growing interests in the substances that regulate cellular radiosensitivity as a strategy to increase tumor radiosensitivity. There are reports that HDAC inhibitors and demethylating agents enhance radiosensitivity [9,12-14]. However, not much information is known about the combined effects of HDAC inhibitors and demethylating agents. In this experiment, human colon and breast cancer cell lines were used to determine the effects of the demethylation agent, 5-Aza-2'deoxycytidine (5-aza-DC), and the HDAC inhibitor, sodium butyrate (SB), and the two agents combined on radiosensitivity.

## Materials and methods

### Cell line culture and reagents

Human colon cancer cell lines RKO (ATCC, USA), breast cancer cell line MCF-7 (KCLB, Korea), and normal colon cell line DDC-112 CoN (ATCC) were used. RKO and MCF-7 cell lines were cultivated in Dulbecco's modified Eagle's medium (DMEM)/F12 (Gibco, Invitrogen Corp., San Diego, California, USA) combined with 10% fetal bovine serum and 1% penicillin/streptomycin using a humidified cultivator that maintained 37°C and 5% CO2. The normal cell line was cultivated using the same cultivator in Dulbecco's modified Eagle's medium (DMEM) combined with 10% fatal bovine serum.

After melting 5-Aza-2'-deoxycytidine (Fluka, Sigma-Aldrich chemic GmbH, Riedstr.) in phosphate-buffered saline, and sodium butyrate(Fluka) in sterilized distilled water, they were stored at 20°C and used when needed.

### Radiation

After 1 × 10^6 ^cells from each cell line were cultured for 24 hours in 100 mm culture dishes, they were divided into three groups. Each group was irradiated with 4 Gy, 6 Gy, or 4 Gy plus additional day of 4 Gy and cultured for 24 or 48 hours after irradiation. The medium used was Dulbecco's modified Eagle's medium (DMEM)/F12(Gibco) combined with 10% fetal bovine serum and 1% penicillin/streptomycin.

### Bisulfate modification and methylation-specific PCR

After being treated with 5-Aza-2'-deoxycytidin and sodium butyrate, and after having received radiation for the proper dose and duration, the DNA was extracted using a QIAamp DNA Mini Kit (Qiagen, Gmbh, Hilden, Germany). The procedure of bisulfate modification of genomic DNA was performed as follows.

After denaturing 2 ug of DNA into 2 M NaOH, the DNA was incubated in 30 ul of 10 mM hydroquinone(Sigma-Aldrich, Inc., St. Louis, USA) and 520 ul of 3 M sodium bisulfate (Sigma) for 16 hours at 50°C. Modified DNA was filtered with a Wizard DNA clean-up system (Promega, Madison, Wisconsin, USA) and then denatured again to 3 M NaOH. 3 M NaOH was precipitated in 100% ethanol and 2.5 M ammonium acetate and, then melted in 20 ul of distilled water. AccuPrime SuperMix I (invitrogen, Life Technologies) was used for PCR; Modified genomic DNA 1 ul was amplified. The product was confirmed with 2.5% agarose gel. PCR conditions and primers are given in Tables 1 and 2. The genes used in this study were MINT 1, 2, 31; methylated in tumor, p16; cyclin dependent kinase inhibitor 4a, p14; p-14 alternative reading frame, E-cadherin; epithelial cadherin.

**Table 1:** Conditions of MS-PCR

	**Denaturation**		**Annealing**			**Extension**	
	
	Temp(°C)	Time(min)	Temp (°C) U/M	Time(sec)	Cycles	Temp (°C)	Time(min)
p14ARF	95	5	62/62	30	40	72	7

p16INK4a	95	5	63/63	30	40	72	7

E-cadherin	95	5	55/57	30	40	72	7

MINT1(M1)	95	5	52/52	30	37	72	7

MINT2(M2)	95	5	59/59	30	40	72	7

MINT31(M31)	95	5	62/60	30	38	72	7

**Table 2:** MS-PCR primers of specific genes analyzed in this study

		Sense primer (5'-3')	Antisense primer (5'-3')
p14ARF	M	GTGTTAAAGGGCGGCGTAGC	AAAACCCTCACTCGCGACG
	
	U	TTTTTGGTGTTAAAGGGTGGTGTAGT	CACAAAAACCCTCACTCACAACAA

p16INK4a	M	TTATTAGAGGGTGGGGCGGATCGC	GACCCCGAACCGCGACCGTAA
	
	U	TTATTAGAGGGTGGGGTGGATTGT	CAACCCCAAACCACAACCATA

E-cadherin	M	TTAGGTTAGAGGGTTATCGCGT	TAACTAAAAATTCACCTACCGAC
	
	U	TAATTTTAGGTTAGAGGGTTATTGT	CACAACCAATCAACAACACA

MINT1(M1)	M	AATTTTTTTATATATATTTTCGAAGC	AAAAACCTCAACCCCGCG
	
	U	AATTTTTTTATATATATTTTTGAAGTGT	AACAAAAAACCTCAACCCCACA

MINT2(M2)	M	TTGTTAAAGTGTTGAGTTCGTC	AATAACGACGATTCCGTACG
	
	U	GATTTTGTTAAAGTGTTGAGTTTGTT	CAAAATAATAACAACAATTCCATACA

MINT31(M31)	M	TGTTGGGGAAGTGTTTTTCGGC	CGAAAACGAAACGCCGCG
	
	U	TAGATGTTGGGGAAGTGTTTTTTGGT	TAAATACCCAAAAACAAAACACCACA

### Cell proliferation assay

After 24 hours of seeding of 3 × 10^3 ^cells each DDC-112 CoN, RKO, and MCF7 in a 96-well plate, 5-Aza-2'-deoxycytidin 4 uM, sodium butyrate 1 mM, and a combination of both were added and then cultivated for 48 hours. An assay was done using a cell proliferation kit II(XTT)(Roche Diagnositcs GmbH, Mannheim, Germany).

### Statistical analysis

For comparison of the treatment effect of radiation, the data were converted to a log scale. Then, using SPSS ver. 13.0, the results were compared with ANOVA(Analysis of Variance), and p values less than 0.005 were considered significant. The average and standard deviation were not converted to log scale in the table of statistics; original data's average and standard deviation were documented.

## Results

### Determining radiation dose and culture time

**Figure 1:**
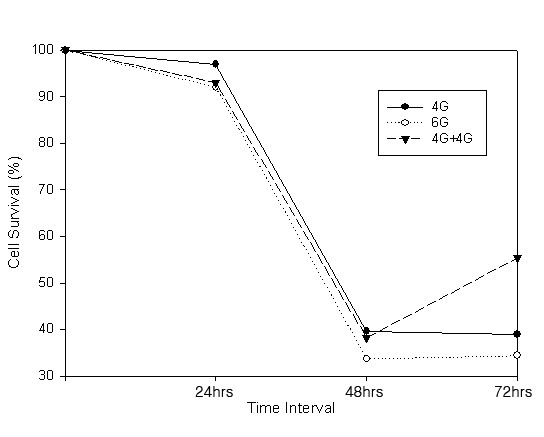
**Cell survival according to different radiation dose(4G, 6G and 4G+4G) and different culture time(24 hrs, 48 hrs and 72 hours)**. There was significant difference in cell survival between 24 hrs and 48 hrs. Also radiation dose 4G and 6G showed more clear survival difference than 4G+4G did.

We irradiated the RKO cell line with the different dose of radiation(4G, 6G, 4G + 4G) and cultured the cells for 24 hours, 48 hours and 72 hours. Then we analyzed the cell survival (Fig 1). For the culture time, there was significant change between day 1 and day 2. But there was no significant change between control and day 1 or between day 2 and day3. For the irradiation dose, 4G and 6G showed more clear survival differences than 4G + 4G did and both 4 Gy and 6 Gy were adequate for analyzing the radiosensitivity. So we chose 4G as irradiation dose and 48 hours as culture time

### CCD-112 CoN, MCF-7 and RKO cell line methylation

In the RKO cell line, all of the tumor suppressor genes were methylated. Half were methylated in the MCF-7 cell line; MINT 1, MINT 31, p16 were methylated and MINT 2, p14, E-cadherin were unmethylated. None were methylated in the CCD-112 CoN cell lines (Table 3).

**Table 3:** The methylation status of each cell lines, CCD-112, MCF-7, RKO

	CCD-112	MCF-7	RKO
MINT1	U	M	M

MINT2	U	U	M

MINT31	U	M	M

P16	U	M	M

P14	U	U	M

E-cadherin	U	U	M

### MS-PCR results after adding 5-Aza-2'-deoxycytidine to the RKO cell line

**Figure 2:**
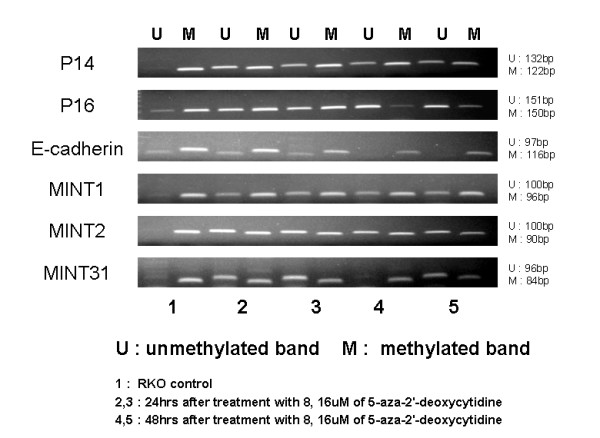
**MS-PCR after 5'-aza-2'-deoxycytidine(5-aza-DC) treatment**. In the control group, most of the genes were methylated, but cell lines treated with 5-aza-DC showed profound increase of demethylated bands.

In the control group, most of the genes were methylated, but cell lines treated with 5-aza-DC showed profound increase of unmethylated bands. (Fig 2).

### MS-PCR results after adding sodium butyrate to the RKO cell line

**Figure 3:**
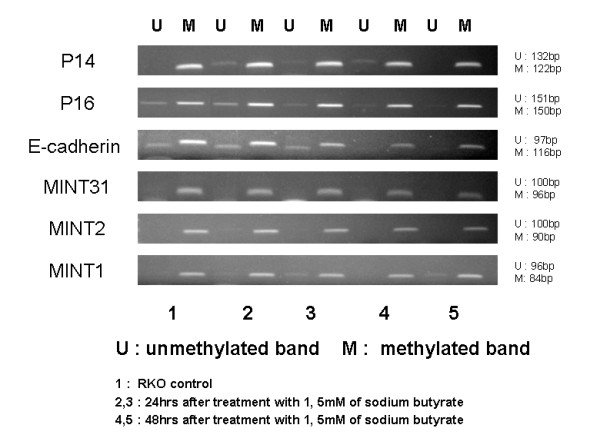
**MS-PCR after sodium butyrate treatment**. Compared to the control group, there were almost no changes in methylation status with the addition of sodium butyrate.

Compared to the control group, there were almost no changes in methylation status with the addition of SB (Fig 3).

### XTT results after addition of sodium butyrate and 5-Aza-2'-deoxycytidine

In the MCF-7 cell line, 87% of the cells survived after radiation alone, 73% after adding 5-aza-DC, and 55.7% after adding SB. Thus both 5-aza-DC and SB increased radiosensitivity, with 5-aza-DC having better results. The combination of the two showed a synergistic effect, which resulted in 45.7% cell survival (p < 0.001).

In the RKO cell line, 56.5% of the cells survived after radiation alone, 47% survived with the addition of 5-aza-DC, and a similar percentage (46%) survived with the addition of SB. The combination of the two resulted in a 39.6% survival rate, showing the synergic effect of the agents (p < 0.001).

**Figure 4:**
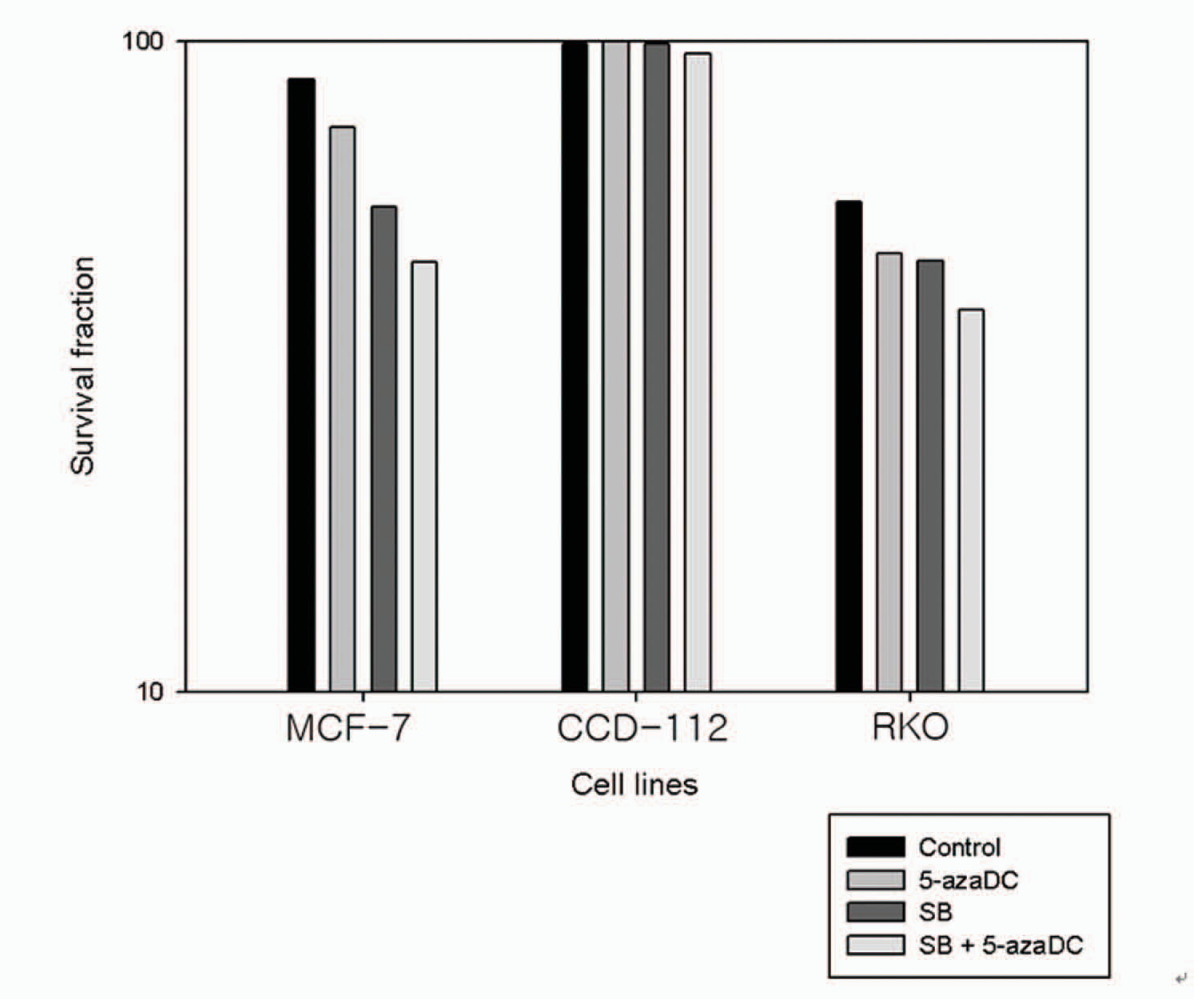
**The effect of 5-azaDC and SB on radiation (logarismic scale)**.

There was no statistical significance among survival rates after treatment with radiation, 5-aza-DC, and SB in CCD-112 CoN cell lines (Table 4, Fig 4).

**Table 4:** The effects of 5-azaDC and SB on radiation

	**Cell Survival %**		
	
With Radiation	MCF-7	CCD-112	RKO
Control	87.2 ± 5.2 (0.97 ± 0.01)	99.1 ± 4.7 (1.00 ± 0.01)	56.5 ± 9.7 (0.87 ± 0.04)

5-azaDC	73.7 ± 9.6 (0.93 ± 0.03)	102.6 ± 3.1 (1.01 ± 0.01)	47.1 ± 4.3 (0.84 ± 0.02)

SB	55.7 ± 5.1 (0.87 ± 0.19)	98.9 ± 10.7 (1.00 ± 0.02)	46.0 ± 3.0 (0.83 ± 0.14)

SB + azaDC	45.7 ± 4.7 (0.79 ± 0.02)	95.8 ± 8.1 (0.99 ± 0.19)	38.6 ± 3.61 (0.79 ± 0.02)

P-value	**<0.001**	0.491	**<0.001**

* p-value was calculated with logarism scale

## Discussion

With the development of molecular radiobiology, recent researches has focused on the molecules and processes that influence the response of cells to radiation. Many different kinds of molecules are known to increase radiosensitivity by influencing the procedures of cell cycle check points, DNA repair, gene transcription, and apoptosis. Recently, studies of epigenetic procedures such as histone deacetylation and DNA methylation have been proposed for enhancing the radiosensitivity of tumor cells.

Out of the many demethylating agents and HDAC inhibitors, we chose 5-aza-DC as the demethylating agent and SB as the HDAC inhibitor for our study. 5-aza-DC is a similar molecule to cytidine. Through a covalent bond to DNMT, it decreases the rate of methylation, thus controlling genetic expression. SB is a short-chain fatty acid that targets the activated region of zinc of HDAC. It has a very short half-life [15].

Histone plays an important role in post-translational modification carried out by histone acetyltransferase and HDAC. Oncogenesis is related to inactivation of histone acetyltransferase, and it is thought that hyperactivation of HDAC suppresses the transcription of tumor suppressor genes, therefore playing an important part in carcinogenesis [16]. Hypoacetylation of histone is related to the structure of condensed chromatin; in this status, transcription is inhibited. Hyperacetylation, on the other hand, creates an open chromatin structure and transcription becomes activated [17]. Inhibition of HDAC is known to increase the radiosensitivity of tumor cells [9,11,13,18,19]. In 1985, Arundel et al [19] reported that SB, an HDAC inhibitor, at a dose relatively without toxicity, enhanced radiosensitivity in colon cancer cell lines. Camphausen et al [18] also reported that MS-275, an HDAC inhibitor, increased radiosensitivy in prostate cancer cell lines. In this experiment, RKO cell lines showed a 56% survival rate with radiation alone, while with SB, 47% survived. In MCF-7 cell lines, radiation alone led to a 87% survival rate, while when radiation was combined with SB, 56% of cells survived, which proved that SB increased radiosensitivity in both RKO and MCF-7 cell lines.

There have been many hypotheses proposed for how HDAC inhibitors enhances radiosensitivity. First, the chromatic compaction has an important role in radiosensitivity, and according to the degree of compaction, chromatin can be divided into euchromatin and heterochromatin. Euchromatin is at a relaxed state in which genes are actively undergoing transcription. Heterochromatin contains inactivated genes, which, is at a highly organized state. Genes with ongoing active transcription are generally more sensitive to radiation, while when chromatin condenses into a highly organic structure where transcription is inactive, DNA becomes protected from double strand breaks(DSB) and resistant to the effect of radiation. Euchromatin contains histones, which are acetylated and phosphorylated, while heterochromatin contains deacetylated and methylated histones [9,20,21]. HDAC inhibitors can change heterochromatin into a euchromatin state, and this mechanism is probably involved in enhancing sensitivity to radiation. Repair of DNA-DSB is another important factor in determining radiosensitivity, and recently, studies have shown that inhibition of DSB repair is the mechanism for increased radiosensitivity with HDAC inhibitors. Expression of γH2AX is an important marker in DSB created by ionizing radiation. When an HDAC inhibitor is used, γH2AX expression is prolonged, and DSB repair is impeded by HDAC inhibitors [13,22]. Chinnaiyan et al [23] reported that HDAC inhibitors take part in down-regulation of the enzymes, DNA-PK and Rad51, which participate in the recovery of DSB, and this DSB recovery plays an important role in determining radiosensitivity.

Hypermethylation of DNA is found commonly in tumor cells, and it suppresses the function of genes that participate in tumor suppression or control the cell cycle, apoptosis or DNA repair [2-4]. Recent studies have shown that demethylating agents enhance radiosensitivity. Dote et al [14] reported that the DNA methylation inhibitor, zebularine, increased the radiosensitivity of tumor cells in vivo and in vitro and that the number of γH2AX foci increased considerably. Our experiment showed that when the demethylating agent 5-aza-DC was added to hypermethylated RKO cells, an unmethylated band was shown on MS-PCR, and both MCF-7 and RKO cell lines showed enhanced radiosensitivity. Another mechanism for the increase in radiosensitivity caused by 5-aza-DC is reported by Takeayashi et al [24]; 5-aza-DC can bring about the hyperacetylation of histones regardless of DNA methylation. Also, there are some reports that demethylating agents interfere with DNA repair [14].

In RKO cell lines, the effect of SB was similar to that of 5-aza-DC, while in MCF-7 cell lines, SB was more effective compared to 5-aza-DC. The function of HDAC inhibitor is considered to be related with the methylation level of the genes. Cameron et al [25] reported HDAC inhibitor Trichostatin A(TSA) could not upregulate the expression of MLH1, TIMP3, CDKN2A which is highly methylated but TSA upregulated the expression of non-methylated CDKN2B. Shen et al [11] also reported that the pathway of histone deacetylation plays a major role when the methylation of the promoter region is at low density. Almost the entire promoter regions of the genes of RKO cell lines were methylated, while about half were methylated in MCF-7 cell lines. This might be the reason why MCF-7 cell lines are more susceptible to HDAC inhibitor than RKO cell lines. Histone deacetylation and DNA methylation are not independent epigenetic mechanisms; they have a very close relationship and influence each other.

There are reports that HDAC inhibitors and demethylating agents have a synergic effect [7,11,25,26]. Cameron et al [25] reported the synergic effect of a HDAC inhibitor, TSA, and a demethylating agent, 5-aza-DC, in re-expression of genes in RKO cell lines. Shen et al [11] also reported that demethylation of the RASSF1α gene and re-expression of mRNA was increased more with a combination of 5-aza-DC and SB compared to using 5-aza-DC alone. In our experiment, the combined effect of 5-aza-DC and SB was superior in enhancing radiosensitivity compared to the use of each agent alone in both MCF-7 and RKO cell lines. The mechanism explaining why the combination effect is better seems to be as follows. DNA methylation recruits HDAC through DNMTs or methylated DNA binding proteins and facilitates histone deacetylation [27,28]. HDAC reinforces DNA methylation through histone H3 lys9 methyltransferase. HDAC and DNA methylation form a loop and influence each other, thus enforcing them [28]. Therefore, through HDAC inhibitor and demethylating agents, the DNA methylation and histone acetylation becomes inactivated and a synergic effect occurs. Also, the combination of SB and 5-aza-DC facilitates the transformation of chromatin into an activated state [8].

There are some reports that 5-aza-DC or SB increase the radiosensitivity in other field than colon or breast cancer. De Schutter et al [29] reported 5-aza-DC with or without TSA could increase radiosensitivity in head and neck squamous cell carcinoma cell line and Camphausen et al [18] also reported MS-275 could increase radiosensitivity in prostate cancer and glioma cell line.

In this experiment, the survival rates of RKO and MCF-7 cell lines after irradiation showed significant differences. One limitation of this experiment is that the found in where effect of 5-aza-DC and SB were not measured under the equal conditions.

## Conclusion

5-aza-DC and SB enhanced radiosensitivity in MCF-7 and RKO cell lines. In RKO cell lines, which are in a relatively hypermethylated state, the effect of 5-aza-DC was similar to that of SB; in MCF-7 cell lines, the effect of SB was better than that of 5-aza-DC. In both cell lines, the combined effect of a demethylating agents, and an HDAC inhibitor showed better results than the effect of each agent used alone. However, this experiment was performed in vitro, and further investigation in vivo is needed.

## Abbreviations

5-aza-DC: 5-aza-2'-deoxycytidine; DSB: double strands break; HDAC: histone deacetylase inhibitor; SB: sodium butyrate; TSA: Trichostatin A.

## Competing interests

The authors declare that they have no competing interests.

## Authors' contributions

CH designed this study and revised manuscript; HJC analyzed the data and wrote the paper; SYK corrected the manuscript; KHK and WKK Collected data; JIK and STO conducted this experiment and JSK helped to design study model.

All authors read and approved the final manuscript.
